# Semaphorin 3A Contributes to Distal Pulmonary Epithelial Cell Differentiation and Lung Morphogenesis

**DOI:** 10.1371/journal.pone.0027449

**Published:** 2011-11-11

**Authors:** Patrice M. Becker, Tracy S. Tran, Michael J. Delannoy, Chaoxia He, John M. Shannon, Sharon McGrath-Morrow

**Affiliations:** 1 Division of Pulmonary and Critical Care Medicine, Department of Medicine, Johns Hopkins University School of Medicine, Baltimore, Maryland, United States of America; 2 The Solomon H. Snyder Department of Neuroscience, Johns Hopkins University School of Medicine, Baltimore, Maryland, United States of America; 3 Cell Biology Imaging Facility, Johns Hopkins University School of Medicine, Baltimore, Maryland, United States of America; 4 Division of Pediatric Pulmonary Medicine, Department of Pediatrics, Johns Hopkins University School of Medicine, Baltimore, Maryland, United States of America; 5 Department of Pediatrics, Childrens Hospital Medical Center, Cincinnati, Ohio, United States of America; University of Giessen Lung Center, Germany

## Abstract

**Rationale:**

Semaphorin 3A (Sema3A) is a neural guidance cue that also mediates cell migration, proliferation and apoptosis, and inhibits branching morphogenesis. Because we have shown that genetic deletion of *neuropilin-1*, which encodes an obligatory Sema3A co-receptor, influences airspace remodeling in the smoke-exposed adult lung, we sought to determine whether genetic deletion of *Sema3A* altered distal lung structure.

**Methods:**

To determine whether loss of *Sema3A* signaling influenced distal lung morphology, we compared pulmonary histology, distal epithelial cell morphology and maturation, and the balance between lung cell proliferation and death, in lungs from mice with a targeted genetic deletion of *Sema3A* (*Sema3A^-/-^*) and wild-type (*Sema3A^+/+^*) littermate controls.

**Results:**

Genetic deletion of *Sema3A* resulted in significant perinatal lethality. At E17.5, lungs from *Sema3A^-/-^* mice had thickened septae and reduced airspace size. Distal lung epithelial cells had increased intracellular glycogen pools and small multivesicular and lamellar bodies with atypical ultrastructure, as well as reduced expression of type I alveolar epithelial cell markers. Alveolarization was markedly attenuated in lungs from the rare *Sema3A^-/-^* mice that survived the immediate perinatal period. Furthermore, Sema3A deletion was linked with enhanced postnatal alveolar septal cell death.

**Conclusions:**

These data suggest that Sema3A modulates distal pulmonary epithelial cell development and alveolar septation. Defining how Sema3A influences structural plasticity of the developing lung is a critical first step for determining if this pathway can be exploited to develop innovative strategies for repair after acute or chronic lung injury.

## Introduction

The semaphorins are a family of evolutionarily conserved secreted and transmembrane proteins that participate in diverse biological processes, including central and peripheral nervous system development and regeneration, cardiovascular, renal and olfactory morphogenesis, immune system function, and cancer progression [1], [2], [3], [4], [5], [6]. Class 3 semaphorins comprise a subfamily of 7 secreted proteins (3A-3G) best characterized as chemorepellants for growing neurons and axons. More recently it has been recognized that semaphorin 3 family members participate in a wide range of neuronal and non-neuronal processes in addition to the cytoskeletal remodeling involved in axonal pathfinding (reviewed in [7]). Semaphorin 3A (Sema3A) was the first identified vertebrate semaphorin, and has been extensively studied as a repulsive axon guidance cue [8], [9], [10], [11] . Sema3A also influences cortical dendritic morphology [3], [12] and neuronal migration [13], as well as apoptosis and proliferation of multiple cell types [14], [15], [16], [17]. Most of the neuronal effects of Sema3A are transduced by a holoreceptor complex, in which an obligatory co-receptor, neuropilin-1 (Nrp-1), functions as the ligand-binding subunit, and signaling occurs through activation of class A plexin receptor family members. Cell type-specific expression of different Sema3A receptor complexes is a key determinant of how this guidance cue exerts selective effects on cellular morphology.

Both Sema3A and Nrp-1 are expressed in fetal, neonatal, and adult lung, yet data regarding how Sema3A signals influence lung morphology and function, or lung structural maintenance in response to injury, are scant. Studies published several years ago suggested that Sema3A signaling through Nrp-1 attenuated branching morphogenesis of fetal lung explants maintained in culture [18]. We recently demonstrated that cigarette smoke-induced airspace enlargement and alveolar epithelial cell death is potentiated by conditional deletion of pulmonary epithelial *Nrp-1* in the lungs of adult animals [19]. These findings led us to hypothesize that Sema3A might be an essential mediator of distal airspace homeostasis.

To test this hypothesis, we evaluated the distal lung morphology of mice with a targeted genetic deletion of *Sema3A* (*Sema3A^-/-^*), maintained on a *C57B/6* genetic background [20]. This strain of mice was initially reported to show no significant embryonic or early postnatal mortality despite severe abnormalities in peripheral nerve projection, although *Sema3A^-/-^* mice independently generated on the *sv129* strain background died within a few days of birth, and the rare survivors exhibited right ventricular hypertrophy and right atrial dilatation [21]. In this study, we demonstrate that the absence of Sema3A was associated with significant perinatal lethality. During late embryonic development, maturation and/or differentiation defects of distal lung epithelium were observed in *Sema3A^-/-^* mice, and the rare *Sema3A^-/-^* mice surviving to postnatal day 14 (P14) or beyond exhibited profound developmental emphysema. Taken together, these data suggest that Sema3A is a critical determinant of distal lung morphogenesis.

## Methods

### Mouse generation and genotyping

Animal studies were approved by the Johns Hopkins Animal Care and Use Committee (protocol number MO10M66). Mouse breeding was performed in central animal facilities. *Sema3A^-/-^* animals on a C57B/6 background were generated by mating *Sema3A^+/-^* mice [20]. Genotyping was performed by tail snip and PCR amplification of tail lysates, using standard techniques and previously reported primers [20]. The morning when a vaginal plug was observed was designated as embryonic day (E) 0.5, and the day of birth as postnatal (P) day 0.

### Lung histology and immunohistochemistry

Embryonic lungs were fixed by submersion in 4% paraformaldehyde overnight at 4^o^C. Postnatal animals were anesthetized, the trachea was cannulated, then the lungs were inflated for histology and immunohistochemical analysis. Lung inflation was performed with 0.5% low-melting agarose at a constant pressure of 25 cm H_2_O, as previously described [19]. The lungs were then fixed *in toto* in 4% paraformaldehyde overnight and subsequently embedded in paraffin. Five µm sections were stained with hematoxylin and eosin, or Periodic Acid-Schiff (PAS) stain for cellular glycogen.

Quantitative lung morphometry was performed as previously described [22]. Briefly, random H&E stained lung sections were photographed with a 10x objective (Nikon Instruments Inc., Melville, NY). Mean linear intercept (MLI) was measured using NIS-Elements AR (Nikon Instruments Inc., Melville, NY).

To localize expression patterns of endogenous Sema3A protein, a placental alkaline-phosphatase (AP)-Nrp-1 ectodomain fusion protein (AP-Nrp1^ecto^; 4 nM) was bound to tissue sections from *Sema3A^+/+^* (wild-type) and *Sema3A^-/-^* (null) mice, using previously reported methods [23]. This probe recognizes endogenous Nrp-1 ligands in the tissue sections. Specificity of AP-Nrp1^ecto^ binding to Sema3A was determined by comparing the binding patterns in lung sections from *Sema3A^+/+^* and *Sema3A^-/-^* mice. Nrp-1 protein expression was determined by hybridizing tissue sections with an AP-Sema3A fusion protein which recognizes endogenous Sema3A receptor complexes. Because Nrp-1 is the ligand binding subunit of the *Sema3A* receptor complex, it is presumed that staining for AP-Sema3A binding primarily reflects sites of endogenous Nrp-1 expression. Nrp-1 protein expression was also evaluated by immunohistochemical staining with an anti-rat Nrp-1 antibody (1∶500, R&D Systems, Minneapolis, MN).

Distal lung epithelial cells were identified using pro-SPC (1∶500; Seven Hills Bioreagents, Cincinnati, OH) or T-1-alpha antibodies (1∶200, DSHB, Iowa City, IO). Determination of cell turnover was evaluated by immunostaining lung sections with an antibody recognizing proliferating cell nuclear antigen (PCNA; 1∶50; Santa Cruz Biotechnology, Santa Cruz, CA), or terminal deoxynucleotide nick-end labeling (TUNEL) of sections for DNA fragmentation (Promega, Boston, MA). Quantification of PCNA and TUNEL positive cells to generate cell proliferative and death indices was performed as previously described [19].

In additional groups of E17.5 *Sema3A^+/+^* and *Sema3A^-/-^* mice, the right upper lobe was snap frozen in liquid nitrogen for subsequent RNA extraction [24], and the left lung was snap frozen in liquid nitrogen for subsequent protein extraction [19]. The right lower lobe was processed for analysis by transmission electron microscopy (TEM). For TEM, embryonic lung lobes were isolated, submerged briefly (30–45 min, 4°C) in fixative containing 2% paraformaldehyde and 2% glutaraldehyde in 0.05M sodium cacodylate buffer with 3mM CaCl_2_ (360mOsm), pH 7.2, then cut into 2 mm cubes and submerged in fresh fixative overnight at 4°C. Following buffer rinse with the addition of 0.18 M sucrose (350 mOsm), samples were post-fixed in 2% osmium tetroxide in 0.1 M sodium cacodylate, 0.09 M sucrose, 3 mM CaCl_2_ (350 mOsm) for 1 hr on ice in the dark. Samples were dehydrated through a graded series of ethanol to 100%, transferred through propylene oxide then embedded in Eponate 12 (Pella) and cured at 60°C for two days. Sections were cut on a Riechert Ultracut E with a Diatome Diamond knife. Eighty nm sections were picked up on formvar coated 1×2 mm copper slot grids and stained with uranyl acetate followed by lead citrate. Some sections were treated for 30 min with 1% aqueous low molecular weight tannic acid (Malinkrodt; after filtration through a 0.22 µm syringe filter), then rinsed with water prior to uranyl acetate staining. Grids were viewed on a Hitachi 7600 TEM operating at 80 kV, and digital images were captured with an AMT 1 K x 1 K CCD camera.

### RNA, protein and satPC expression in lung tissue homogenate

Quantitative real time PCR (qPCR) of Sema3A RNA isolated from lung homogenate was performed using previously described methods [24], after isolation of RNA from frozen tissue samples (SV Total RNA Isolation Kit; Promega, Boston, MA), and conversion to cDNA (iScript cDNA Synthesis Kit; BioRad, Hercules, CA). qPCR was performed was performed using TaqMan probes and primer sets (Applied Biosystems), with *Actb* as an internal control. The PCR reactions and relative quantifications were performed using 25 ng of cDNA per reaction on a 7300 Real-Time PCR system (Applied Biosystems).

Expression of Aquaporin-5 (Aqp5), and T1-alpha protein were determined by Western blot analysis of whole left lung homogenate form E17.5 mice, using methods previously described [19]. Primary antibodies for T1-alpha were purchased from The Developmental Studies Hybridoma Bank, and Aqp 5 antibodies were a generous gift from Ramana Sidhaye. Protein expression was quantified using densitometry, and normalized to expression of GAPDH on the same membranes. Disaturated phosphatidylcholine (SatPC) levels were measured in whole lung homogenate from E18.5 mice, using methods previously described [25].

### Statistical analysis

Statistical analysis was performed using the StatView program (SAS Institute). To determine the age-dependent effects of Sema3A deletion on body weight, MLI, and cell proliferative and death indices, comparisons between groups were made using two-way ANOVA (genotype by age). If significant interactions (p≤0.05) were identified, least-significant differences were calculated to allow comparison of individual group means. Differences in lung homogenate expression of T1-alpha and Aquaporin-5 protein were determined using non-parametric analysis (Mann-Whitney) of densitometric data.

## Results

### Targeted genetic deletion of Sema3A reduces viability

As was previously reported [20], in our hands the *Sema3A^-/-^* mice were born from heterozygous matings at expected Mendelian ratios. Complete genotype analysis was performed on offspring from 15 matings between *Sema3A^+/-^* mice. Twenty-two percent (29/129) of pups were wild type (*Sema3A^+/+^*), 54% (70/129) were heterozygous (*Sema3A^+/-^*), and 23.2% (30/129) were homozygous null (*Sema3A^-/-^*). These ratios were similar for 7 litters harvested at E17.5, suggesting that fetal viability is not affected by loss of Sema3A. However, almost two-thirds of *Sema3A^-/-^* mice died during the first postnatal day, and only rare survivors lived to P14 or beyond. No discernible differences in body weight were noted for the subset of surviving *Sema3A^-/-^* mice in the immediate postnatal period (Table 1). However, some newborn *Sema3A^-/-^* animals were dead prior to observation. The few *Sema3A^-/-^* animals surviving beyond P1 generally became distinguishable from their littermates between P7 and P14 on the basis of smaller body size. It was sometimes possible to predict impending late postnatal mortality of a *Sema3A^-/-^* pup because of increasingly labored respiration for approximately 24 hrs prior to death.

**Table 1:** Mouse body weights.

	P1	P14	P28
***Sema3A^+/+^***	1.48 ± 0.09g(n = 6)	7.47 ± 0.29g(n = 4)	14.92 ± 0.36g(n = 11)
***Sema3A^+/-^***	1.44 ± 0.03g(n = 29)	6.18 ± 0.38g *(n = 18)	12.73 ± 0.71g *(n = 6)
***Sema3A^-/-^***	1.34 ± 0.04g(n = 5)	4.21 ± 0.32g * ^,^ **(n = 10)	5.78 + 0.53g * ^,^ **(n = 8)

*p<0.0001 vs *Sema3A^+/+^*.

**p<0.0001 vs *Sema3A^+/-^*.

**Figure 1:**
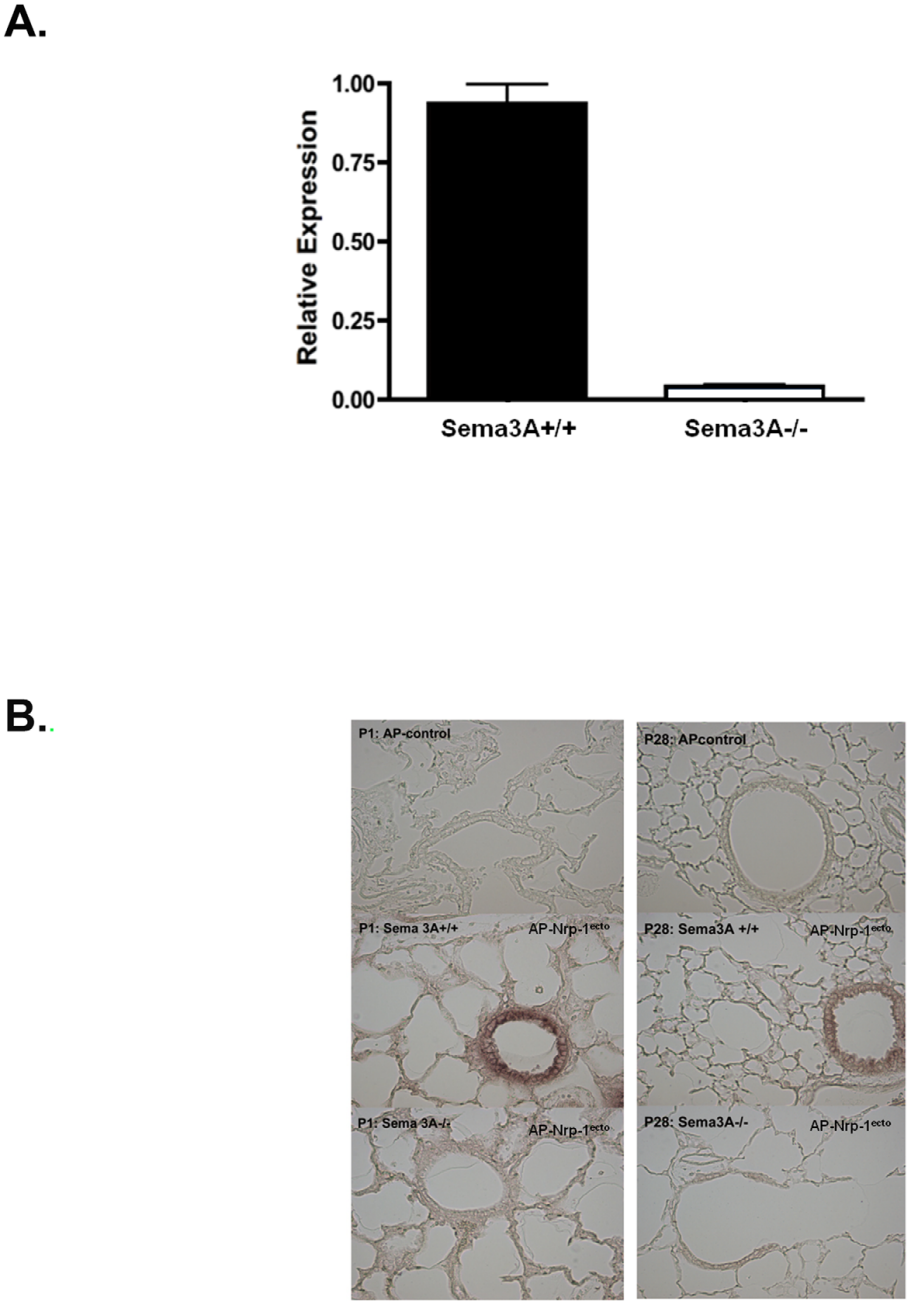
Sema3A expression. **A.** Reduced Sema3A expression in the lungs of *Sema3A^-/-^* mice was assessed using qPCR. **B.** Representative AP-Nrp-1^ecto^ binding in tissue sections from *Sema3A^+/+^* (middle panels) and *Sema3A^-/-^* mice (bottom panels) at P1 (left) and P28 (right). Prominent AP-Nrp-1^ecto^ binding was seen in bronchiolar epithelium of littermate control (*Sema3A^+/+^*) mice at both P1 and P28. This pattern of tissue binding of AP-Nrp1^ecto^ was absent in lung sections from *Sema3A^-/-^* animals, suggesting specificity of the signal for Sema3A expression. Top panels represent non-specific AP staining in lung sections hybridized with a control AP-vector. (Magnification  =  20x).

**Figure 2:**
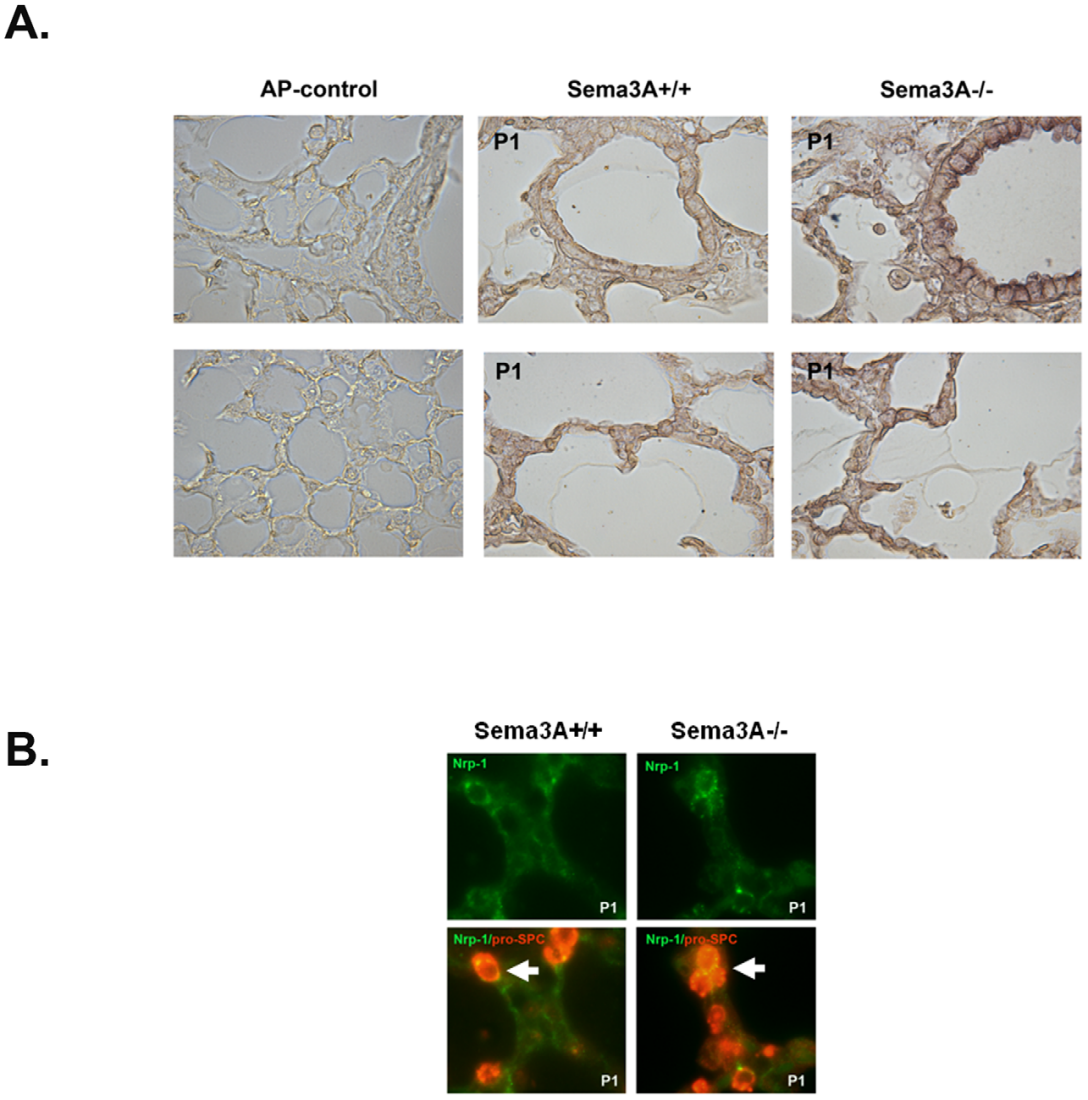
Nrp-1 expression. **A.** Representative AP-Sema3A binding in tissue sections from *Sema3A^-/-^* (right panels) and *Sema3A^+/+^* littermate control (left panels) mice at P1. (Magnification  =  20x) **B.** Representative dual immunostaining for Nrp-1 (green) and pro-SPC (red) in lungs from *Sema3A^-/-^* (right panels) and *Sema3A^+/+^* (left panels) mice. A subset of Nrp-1 expressing cells stain positive for the ATII marker, pro-SPC (white arrows). (Magnification  =  60x).

Shown in Figure 1A, qPCR demonstrated negligible amounts of Sema3A mRNA in lung homogenate from *Sema3A^-/-^* animals as compared with wild type (*Sema3A^+/+^*) littermate controls. Postnatal pulmonary Sema3A protein expression, assessed by AP-Nrp1^ecto^ binding to tissue sections, was largely confined to small airway epithelium in *Sema3A^+/+^* mice. Expression was greatest at P1, but was sustained through P28 (Figure 1B). This pattern of AP-Nrp1^ecto^ binding was absent in lung sections from *Sema3A^-/-^* animals, suggesting specificity of the probe for Sema3A rather than another Nrp-1 ligand. Expression of Nrp-1, the Sema3A obligate co-receptor, was determined by tissue binding of AP-Sema3A. AP-Sema3A binding was seen in cells throughout alveolar septae as well as in bronchiolar epithelium at P1 (Figure 2A). Immunostaining suggested that some of the septal cells which express Nrp-1 were of epithelial origin, demonstrated by co-labeling with Nrp-1 and pro-SPC antibodies (Figure 2B).

### *Sema3A* deletion disrupts distal lung morphology

**Figure 3:**
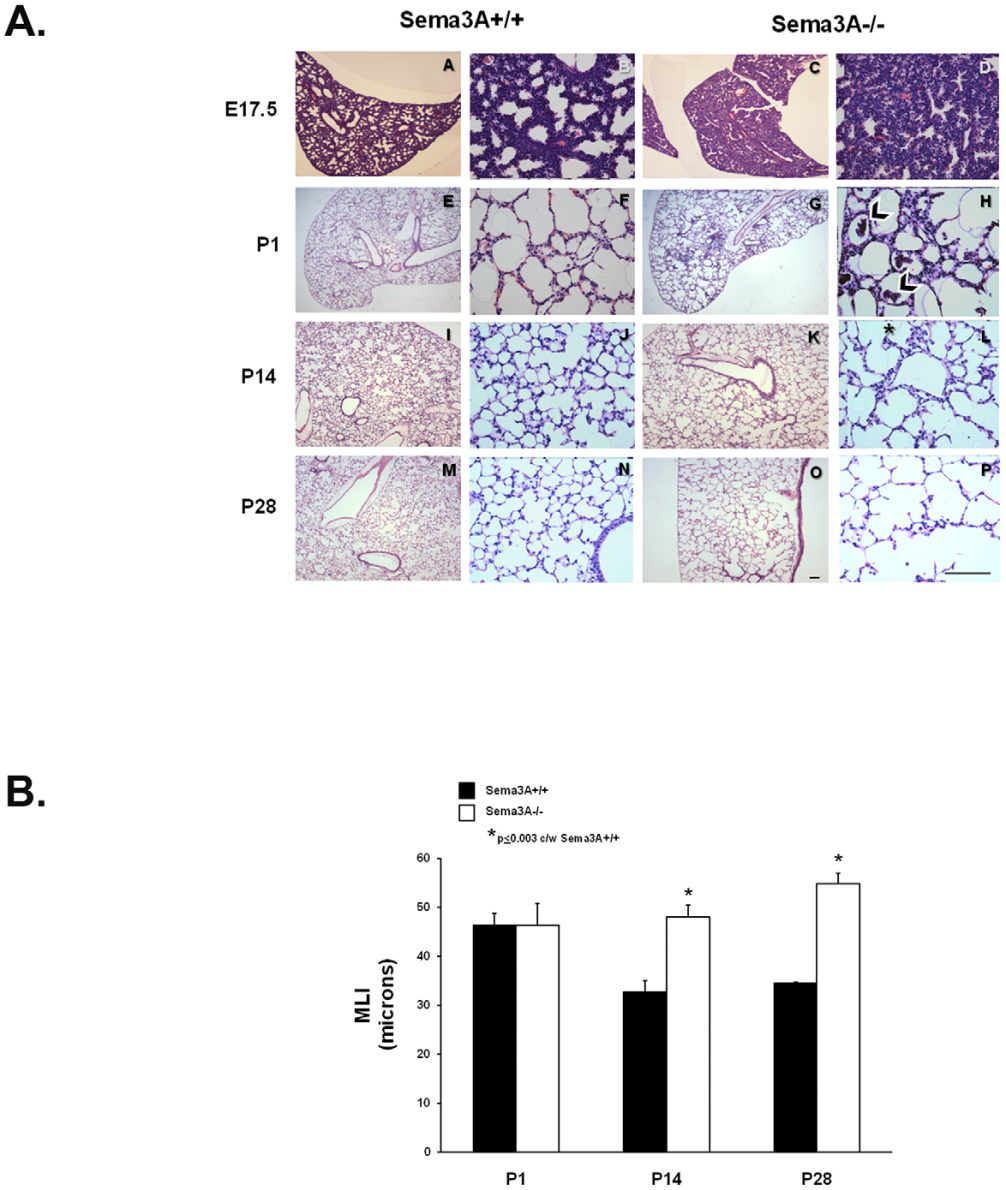
Lung morphology is abnormal in *Sema3A^-/-^* mice. **Top panel.** Representative lung histology from *Sema3A^-/-^* mice (right panels) and *Sema3A^+/+^* littermate controls (left panels) at E17.5, P1, P14 and P28. Prominent septation defects were seen in all *Sema3A^-/-^* mice that survived to P14 or beyond. At the earlier ages, septae of lungs from many *Sema3A^-/-^* mice were thickened, and clusters of cellular debris were sometimes detected in airspaces at P1 (black arrowheads in panel H). Scale bar represents 50 µm. **Bottom panel.** Airspace size was quantitated morphometrically by calculation of the mean linear intercept (MLI). MLI increased in all *Sema3A^-/-^* mice (white bars) surviving to P14 or beyond as compared with *Sema3A^+/+^* littermate control animals (black bars) and *Sema3A^+/-^* littermates (see text). Data represent mean ± SE for 4 mice/group at each time point.

We next evaluated lung histology of *Sema3A^-/-^* pups and found that it was abnormal at all ages examined (E17.5, P1, P14 and P28) (Figure 3). Lungs from E17.5 *Sema3A^-/-^* mice had reduced airspace and thickened septae. In surviving *Sema3A^-/-^* mice, cellular debris was sometimes seen in the airspaces at P1 (Figure 3H, black arrowheads). Profound airspace enlargement, quantified by morphometric estimation of the mean linear intercept (MLI), was seen in the few *Sema3A^-/-^* mice that survived to P14 or beyond, when compared with both *Sema3A^+/+^* (bar graph, Figure 3) and *Sema3A^+/-^* littermates. Because surviving *Sema3A^-/-^* mice were significantly smaller than their littermate controls at the later postnatal ages, we cannot definitively exclude the possibility that nutritional deficiencies contributed to attenuation of postnatal alveolarizaton. However, the feeding and nesting behavior of all neonatal mice we observed did not differ appreciably among littermates. Furthermore, MLI estimated in lungs harvested from *Sema3A^+/-^* mice (44.1+1.2, 32.6+2.1, and 33.6+1.0 microns at P1, P14, and P28, respectively) did not differ from age-matched *Sema3A^+/+^* animals, despite their smaller body size.

### *Sema3A* deletion is associated with enhanced cell death in postnatal mice

**Figure 4:**
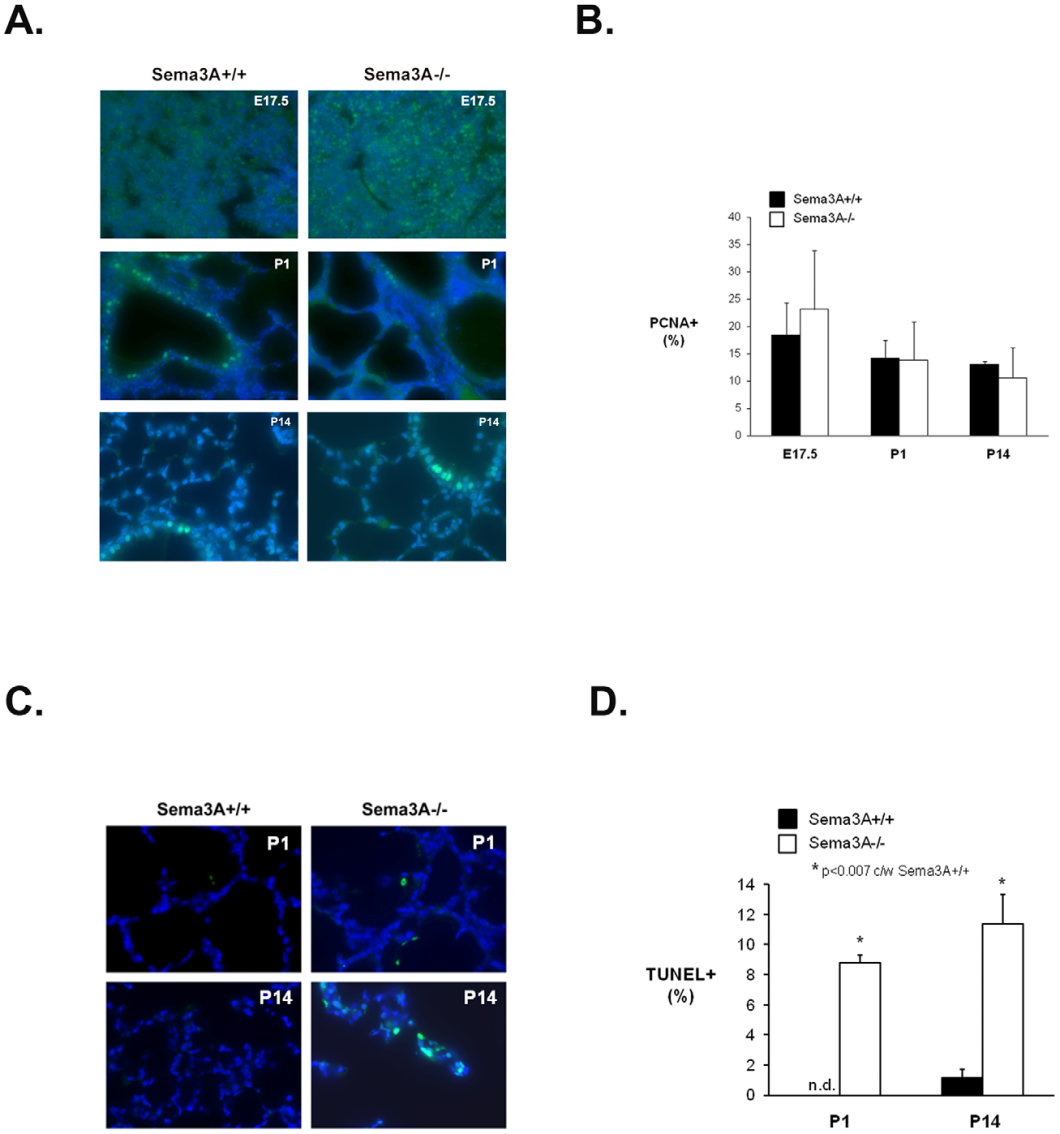
Lung cell death but nor proliferation is altered in *Sema3A^-/-^* mice. **A.** Widespread immunostaining for the proliferative marker PCNA (green) was seen in lungs from both E17.5 *Sema3A^+/+^* and *Sema3A^-/-^* mice (top panels). By P1 (middle panels) and P14 (lower panels), PCNA staining was largely confined to cells lining small airways, and did not differ between *Sema3A^-/-^* animals and littermate controls. Nuclei are counterstained with DAPI (blue) (Magnification = 40x). **B.** Shown graphically, the average proliferative index did not significantly differ between *Sema3A^-/-^* (white bars) and *Sema3A^+/+^* (black bars) mice at any time point evaluated (bars represent mean ± SE for n = 3 animals/group). **C.** Representative TUNEL labeling (green) in lungs from *Sema3A^-/-^* mice surviving to P1 (top right panel) and P14 (bottom right panel). Shown for comparison in the left panels are lungs from *Sema3A^+/+^* littermate controls. Nuclei are counterstained with DAPI (blue). (Magnification  = 40x). **D.** Apoptotic index increased significantly in *Sema3A^-/-^* animals (white bars) as compared with *Sema3A^+/+^* littermate control mice (black bars) at both P1 and P14 (n.d. =  not detected; bars represent mean ± SE for n = 3 animals/group).

In order to determine whether the widened septae and reduced airspace seen in *Sema3A^-/-^* lungs during late embryogenesis were due to altered cell turnover, immunostaining for PCNA and TUNEL labeling of tissue sections were performed. Shown in Figure 4A, widespread immunostaining for PCNA was seen in E17.5 lungs from *Sema3A^+/+^* littermate control and *Sema3A^-/-^* mice. By P1 and P14, PCNA positive cells were largely confined to airways, and the number and pattern of PCNA positive cells appeared similar between surviving *Sema3A^-/-^* mice and *Sema3A^+/+^* littermate controls. Proliferative index was not significantly altered by Sema3A deletion at any age point examined (Figure 4B). In contrast, rare TUNEL positive cells were seen in E17.5 lungs from mice of either genotype (data not shown), but the cell death index (Figure 4D) was significantly enhanced in lungs from the few *Sema3A^-/-^* animals surviving birth, with TUNEL positive cells present within alveolar septae and along secondary crests (Figure 4C).

### Pulmonary epithelial cell maturation is impaired in *Sema3A^-/-^* mice

**Figure 5:**
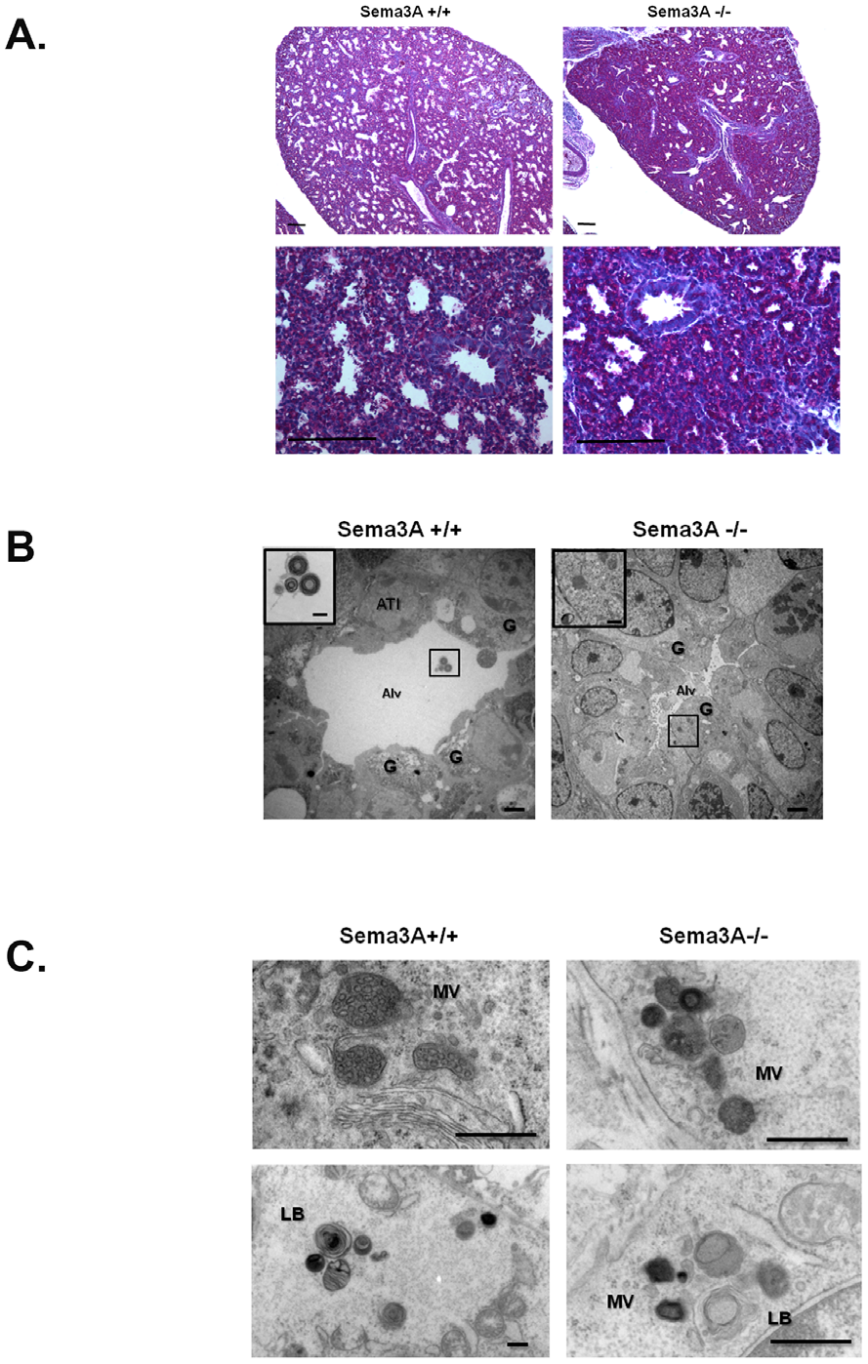
Alveolar epithelial cell morphology is altered in *Sema3A^-/-^* mice. **A.** PAS staining was increased in lungs from E17.5 *Sema3A^-/-^* mice (right panels) as compared with *Sema3A^+/+^* littermate controls (left panels). Bar  =  10 µ. Results are representative of 3 animals/group. **B.** TEM confirmed reduced distal airspace size (Alv) and the presence of fewer ATI in *Sema3A^-/-^* mice (right panel) as compared with *Sema3A^+/+^* littermate controls (left panel). Cytoplasmic glycogen (G) was seen in epithelial cells surrounding distal airspaces (Alv). Lamellar bodies were seen within airspaces (inset) and ATII of *Sema3A^+/+^* animals (left panel), whereas ATII of *Sema3A^-/-^* mice contained primarily multivesicular bodies (inset; right panel). Images captured at 5000x, scale bar  =  2 µ; insets captured at 30,000x, scale bar  =  500nm. Results are representative of 3 animals/group. **C.** TEM images of ATII from lungs of *Sema3A^+/+^* littermate control mice at E17.5 demonstrate normal multivesicular (MV; top right panel) and lamellar (LB; bottom left panel) body formation. In contrast, multivesicular (top right panel) and lamellar (bottom right panel) bodies in ATII from age-matched *Sema3A^-/-^* animals were often smaller and had poorly formed membranous inclusions. Scale bar  =  500 nm in all panels. Results are representative of 3 mice/group.

Because early perinatal respiratory lethality is often related to impaired epithelial maturation, and some of the Nrp-1 cells expressing cells in the lung periphery were of epithelial origin, we sought to determine whether *Sema3A* deletion might alter pulmonary epithelial cell development. Cytoplasmic glycogen is abundant in immature type II alveolar epithelial cells (ATII) cells prior to its utilization for surfactant production. Both PAS staining and TEM demonstrated increased prominence of intracellular glycogen in the distal alveolar epithelial cells of E17.5 *Sema3A^-/-^* mice (Figures 5A and B). In addition, ultrastructural evaluation of ATII in the lungs of *Sema3A^-/-^* animals at E17.5 suggested the presence of fewer multivesicular and lamellar bodies, and those seen were smaller with atypical membranous inclusions when compared with ATII in *Sema3A^+/+^* littermate control lungs (Figure 5C), despite the fact that expression of mRNA for surfactant proteins in E17.5 whole lung homogenate did not differ significantly between *Sema3A^-/-^* mice and littermate control animals (data not shown). As an additional assessment of alveolar epithelial maturity, we measured SatPC levels in lungs from E18.5 *Sema3A^-/-^* and *Sema3A^+/+^* mice, and found no significant differences (3.109±0841 and 3.196±0.2628 µmol SatPC/g lung weight for *Sema3A^-/-^* and *Sema3A^+/+^* mice, respectively), although this does not preclude the possibility that SatPC levels differ at or after birth.

**Figure 6:**
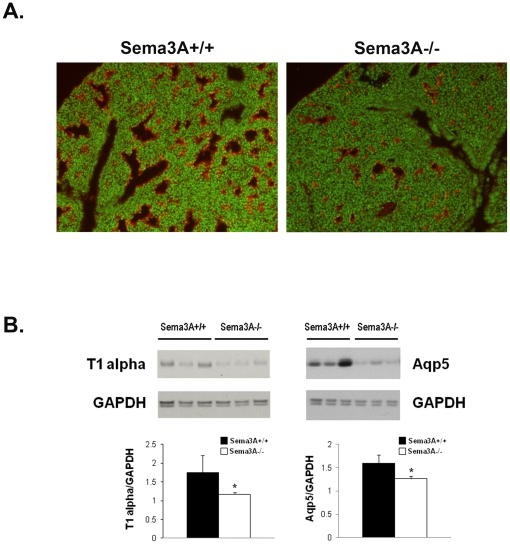
ATI markers are reduced in *Sema3A^-/-^* mice. **A.** Reduced immunostaining for the type I alveolar epithelial cell marker, T1-alpha (red), is seen in lung from a *Sema3A^-/-^* mouse at E17.5 (right) as compared with a *Sema3A^+/+^* littermate control (left). Nuclei are counterstained with Fast Yellow (green). Results are representative of 3 animals/group. (Magnification = 10x). **B.** Western blotting demonstrates decreased expression of both T1-alpha (left) and Aqp5 (right) protein in lung homogenate from *Sema3A^-/-^* animals as compared with *Sema3A^+/+^* littermate controls. Results are representative of 7-9 animals/genotype.

ATII cells are precursors of the Type I alveolar epithelial cells (ATI) required for gas exchange in the maturing distal lung. We therefore compared immunohistochemical expression of the ATI surface protein, T1-alpha, in lungs from *Sema3A^-/-^* mice and *Sema3A^+/+^* littermate controls. Shown in Figure 6A, T1-alpha staining was widespread in cells lining the distal airspaces in *Sema3A^+/+^* lungs at E17.5, but expression appeared attenuated in lungs from *Sema3A^-/-^* animals. Western blotting of whole lung homogenates was used to confirm reduced expression of both T1-alpha and another ATI protein, Aqp-5, in lungs from *Sema3A^-/-^* mice (Figure 6B). Additionally, ATI were seen less frequently by TEM in lung sections from *Sema3A^-/-^* mice, as compared with *Sema3A^+/+^* littermate controls (Figure 5B). Taken together, these data support a role for Sema3A in maturation and/or differentiation of the distal lung epithelium during development.

## Discussion

Although first identified as a mediator of axonal pathfinding during morphogenesis of the peripheral nervous system [20], [21], several studies support a role for Sema3A in development and patterning of non-neuronal tissues, including salivary gland, ureter, and kidney [1], [7], [26], [27], [28]. Prior reports demonstrated that exogenous Sema3A protein attenuated branching morphogenesis of E11.5 fetal lung explants maintained in culture [18], [29], but the role of Sema3A in lung development has not been fully characterized. Using *in vivo* loss of function modeling, we show here that Sema3A signals modulate distal lung epithelial cell development and postnatal alveolarization.

Using a tissue binding assay and an AP-Nrp1^ecto^ probe, our data demonstrate patterns of early postnatal pulmonary Sema3A protein expression that are comparable with previously reported data using *in situ* hybridization [29] or immunohistochemistry [18] for Sema3A localization. Because Sema3A is a secreted protein, we also localized sites of expression for Sema3A receptor complexes, by hybridizing lung tissue sections with an AP-Sema3A fusion protein. The alveolar pattern of AP-Sema3A binding to lung sections harvested from P1 mice was similar to previously reported Nrp-1 gene expression assessed using *in situ* hybridization during late embryogenesis [29]. Our results extend previous observations by demonstrating that a subset of Nrp-1 expressing cells in the lung periphery are of epithelial origin, since they co-express pro-SPC.

Because targeted deletion of *Sema3A* was associated with significant lethality following the transition to air breathing, we evaluated fetal lungs from *Sema3A^-/-^* mice for both structural abnormalities and disruption of distal epithelial maturation and differentiation. Alveolar septae in lungs from *Sema3A^-/-^* animals at E17.5 were thickened with reduced distal airspace. In addition, morphologic characterization suggested incomplete or delayed maturation and/or differentiation of alveolar epithelial cells. Perinatal lethality from respiratory failure has been reported after genetic deletion of the ATI protein T1-alpha, and in several murine models of transgenic misexpression or deletion of transcription factors which disrupt distal pulmonary epithelial cell development [24], [30], [31], [32], [33], [34], [35]. Mortality in these prior reports has been linked with either dysregulation of surfactant gene expression and function [31], [33], [34] or a reduction in Type I alveolar epithelial cell development [30], [32], [35]. It is of interest to note that in at least two of these previously reported models, downregulation of Sema3A expression was detected using microarray analysis of lung tissue homogenate. In the first instance, significantly decreased Sema3A transcript was found after pulmonary misexpression of the epithelial specific Ets transcription factor, ELF5. ELF5 has been reported to repress distal epithelial specification and differentiation in the lung in a manner that is tightly developmentally regulated [24]. More recently, reduced Sema3A gene expression was noted following genetic deletion of the transcriptional regulator, coactivator-associated arginine methyltransferase I (CARM1). The phenotype of late stage fetal lungs from *CARM1* null mice was remarkably similar to what we observed in *Sema3A* null animals, with thickened alveolar walls, and attenuated differentiation of ATI cells [32]. It is perhaps not surprising that the pulmonary phenotype of *Sema3A^-/-^* mice is less severe than what has been reported after disruption of factors that transcriptionally regulate the differentiation of lung epithelium [31], [34], [36], [37], [38]. The finding that not all *Sema3A^-/-^* mice die immediately after birth suggests alternative pathways may compensate for the effects of *Sema3A* deletion on epithelial cell maturation and/or differentiation. This speculation is supported by the variable mortality reported for targeted deletions of *Sema3A* generated in differing genetic backgrounds [20], [21].

In mice, as in humans, the formation of alveolar septae occurs largely during postnatal life [39]. Interestingly, the few *Sema3A^-/-^* mice surviving to P14 exhibited profound airspace enlargement, and MLI remained significantly increased in the rare animals surviving to adulthood. Signaling initiated by Sema3A requires the expression of its obligate co-receptor, neuropilin-1 (Nrp-1). Constitutive genetic deletion of *Nrp-1* in mice results in embryonic lethality by E12.5, thus a definitive requirement for Nrp-1 in alveolar epithelial maturation and differentiation has not been determined. However, we previously reported that conditional deletion of *Nrp-1* in the alveolar epithelium of one week old mice led to airspace enlargement in adulthood [19], supporting a potential role for epithelial Sema3A-Nrp-1 signaling in the processes controlling lung septation. We have not yet determined how disruption of Sema3A-induced distal epithelial cell maturation and/or differentiation might attenuate postnatal alveolar septation, although a recent report by Srisuma et al. suggested that primary abnormalities in ATII may impair epithelial-mesenchymal interactions coordinating elastogenesis and proper airspace formation [40].

Sema3A deletion could also impair postnatal alveolarization by pathways independent of those mediating epithelial differentiation. Nrp-1 expression is not restricted to epithelium, and prior reports demonstrate that Sema3A-Nrp-1 signals regulate angioblast migration and vascular patterning [41], [42], [43], as well as differentiation of vascular precursors to endothelial cells [44]. It has long been recognized that alveolar septation is always associated with capillary invasion, and pulmonary vascular development must match epithelial morphogenesis to ensure optimal gas exchange in the lung. Endothelial Nrp-1 mediates signaling of both Sema3A and vascular endothelial growth factor (VEGF), and altered VEGF expression has been shown to profoundly influence both lung alveolarization and vascularization [45], [46]. Loss of Sema3A could therefore impair distal lung homeostasis by disrupting the finely tuned balance between Sema3A- and VEGF-induced Nrp-1 signals in the developing vasculature. We did not detect significant differences in expression of mRNA for a panel of endothelial genes, including VEGFR2/flk-1, PECAM, flt4, ESAM-1, VEGF-A, or VEGF-C, in lung homogenate from E17.5 *Sema3A^-/-^* animals and *wild-type* littermate controls (data not shown). This suggests that endothelial cell number may not be altered in the absence of Sema3A, but additional experiments will be required to determine whether Sema3A plays a role in vascular patterning, or apposition of the vasculature and maturing alveolar epithelium.

Prior reports demonstrate that Sema3A and Nrp-1 signaling in extra-pulmonary tissues can influence multiple processes that may be critical for lung morphogenesis [39], [47], including cell viability and proliferation, fate specification, motility, and matrix adhesion [6], [14], [15], [16], [17]. Our data are the first to suggest that loss of Sema3A signaling temporally regulates cell death during distal lung development. Because dysregulation of lung structural cell proliferation [48] and death [49], [50], [51] have been shown to play an important role in the pathogenesis of emphysema in experimental models, Sema3A signals may therefore also be important determinants of plasticity following lung injury and alveolar remodeling. Consistent with this hypothesis, we recently demonstrated that conditional deletion of Nrp-1 in the distal lung epithelium of adult mice augmented both epithelial cell death and airspace enlargement in response to chronic cigarette smoke exposure [19].

In summary, these data support a significant role for Sema3A in late fetal and early postnatal lung development. Perinatal lethality of *Sema3A^-/-^* mice was linked with reduced expression of ATI markers, and ATII cells exhibited some features characteristic of impaired maturation, although surfactant production was not grossly impaired in the days prior to birth. Additional studies will be required to determine whether these phenotypic epithelial cell changes are responsible for disruption of alveolar septation in *Sema3A^-/-^* animals. Defining how Sema3A influences structural and functional plasticity of the developing lung is a critical first step for determining if this pathway can be exploited to develop innovative strategies for repair after acute or chronic lung injury.
